# A Machine Learning Model for Torsion Strength of Externally Bonded FRP-Reinforced Concrete Beams

**DOI:** 10.3390/polym14091824

**Published:** 2022-04-29

**Authors:** Ahmed Deifalla, Nermin M. Salem

**Affiliations:** 1 Engineering and Construction Management Department, Future University in Egypt (FUE), Cairo 11835, Egypt; 2 Electrical Engineering Department, Future University in Egypt (FUE), Cairo 11835, Egypt; nfawzy@fue.edu.eg

**Keywords:** torsion, FRP, strengthening, machine learning

## Abstract

Strengthening of reinforced concrete (RC) beams subjected to significant torsion is an ongoing area of research. In addition, fiber-reinforced polymer (FRP) is the most popular choice as a strengthening material due to its superior properties. Moreover, machine learning models have successfully modeled complex behavior affected by many parameters. This study will introduce a machine learning model for calculating the ultimate torsion strength of concrete beams strengthened using externally bonded (EB) FRP. An experimental dataset from published literature was collected. Available models were outlined. Several machine learning models were developed and evaluated. The best model was the wide neural network, which had the most accurate results with a coefficient of determination, root mean square error, mean average error, an average safety factor, and coefficient of variation values of 0.93, 1.66, 0.98, 1.11, and 45%. It was selected and further compared with the models from the existing literature. The model showed an improved agreement and consistency with the experimental results compared to the available models from the literature. In addition, the effect of each parameter on the strength was identified and discussed. The most dominant input parameter is effective depth, followed by FRP-reinforcement ratio and strengthening scheme, while fiber orientation has proven to have the least effect on the prediction output accuracy.

## 1. Introduction

In recent years, there have been more reports of structural failures attributed to torsion [1]. Whittle identified several reasons for failure, which included but were not limited to the following: (1) design errors; (2) structural modeling errors; (3) inappropriate extrapolation of the code of practice; (4) inadequate assessment of critical forces paths. In addition, failure could be because of aging and lack of maintenance. Reinforced concrete (RC) members subjected to large torsion may fail quite suddenly, which is undesirable and needs to be avoided. Thus, analysis of damage to engineering structures, rehabilitation, and strengthening is becoming a necessity. The choice of material used for that purpose is of paramount importance. FRP has many advantages in engineering applications [2,3,4,5,6].

During the last few decades, rehabilitation and strengthening of structures using externally bonded fiber-reinforced polymer (EB-FRP) have been an important research topic worldwide [7,8,9,10]. Although many reinforced concrete (RC) members are subjected to significant torsion, most of the available research investigates concrete-strengthened members’ flexure and shear behavior. The high cost of infrastructure replacement has prompted research into various strengthening and rehabilitation techniques. Torsion strengthening is required in many projects [11]. Structural elements subjected to torsion experience diagonal tension and compression, thus failing in an undesirable brittle manner, leading to inadequate behavior during earthquakes [12,13,14,15,16,17]. Therefore, a simple yet accurate torsion design of concrete structural elements is essential. Much more research studies are required to give physical significance to the torsion design of concrete beams with FRP [18,19,20].

**Figure 1:**
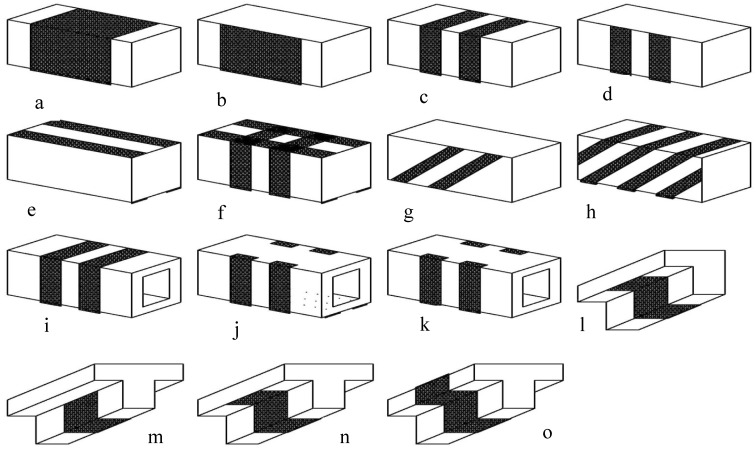
Torsion strengthening schemes using FRP (**a**) Entire beam for rectangular beam; (**b**) U entire beam for rectangular beam; (**c**) 90 strips for rectangular beam; (**d**) U strips for rectangular beam; (**e**) Longitudinal strips for rectangular beam; (**f**) 90 strips and longitudinal strips for rectangular beam; (**g**) 45 strips one side for rectangular beam; (**h**) 45 strips spiral around beam for rectangular beam; (**i**) 90 strips for box beam; (**j**) U anchorage strips and longitudinal strips for box beam; (**k**) U anchorage strips for box beam; (**l**) Extended U entire beam for spandrel beam (with multiple FRP orientations); (**m**) U entire beam for T beam; (**n**) Extended U entire beam for T beam; (**o**) Entire beam for T beam.

Although most of the early research efforts focused on beams strengthened using EB-FRP subjected to moments or shear, more recently, starting in the year 2001, the torsion behavior of concrete beams with EB-FRP has gained much attention. Over the last few years, several experimental investigations worldwide have been conducted to study the torsional behavior of strengthened concrete beams, as shown in Figure 1 [21,22,23,24,25,26]. However, much more work is required to give physical significance to the torsion design of concrete beams strengthened using EB-FRP.

In the case of torsion strengthening, de-bonding failure is the most common failure mode, which is usually accompanied by excessive concrete cracking or bond slippage at the fiber-reinforced polymer (FRP) and concrete interface [27,28,29,30,31]. Available bond models developed based on simple shear testing of FRP sheets bonded to concrete blocks have shown that the FRP ultimate strain will probably not be reached, regardless of how large the interface length between the FRP and the concrete is. Existing models for torsion strength lack accuracy, and thus, a need for reliable and accurate strength models is a mandate.

Machine learning-based models have proven to be reliable in predicting the strength of many problems, including, but not limited to, punching shear and shear of beams [32,33,34]. However, very limited machine learning models were developed for torsion, especially for the torsion of beams strengthened with FRP. Thus, this research study focused on developing a machine learning model for the torsion strength of EB-FRP beams. In this regard, an experimental database was assembled, and the effects of various parameters were investigated. In addition, selected parameters such as FRP reinforcement ratio, fiber orientation, and concrete compression strength were implemented to develop a machine learning model. Moreover, several ML models were created, and their strength was compared with that of the experimental database results and that from the available design models. Finally, the importance of the various variables on the strength was found and discussed. This study could help further design code development.

## 2. Experimental Database Profiles

Since 2001, FRP has been studied as an externally reinforcing material for beams under torsion. Table 1 shows the statistical measures for the collected experimental database. For the case of concrete beams strengthened using EB-FRP, the following parameters were investigated: (1) beam width (b); (2) beam effective depth (d); (3) FRP Young’s modulus (E) as an indication of the type of fiber used, carbon FRP (CFRP) and glass FRP (GFRP); (4) FRP reinforcement ratio (ρ) taken as $\frac{ntw}{t_{c}s}$, where n is the number of layers, t is the thickness of FRP sheets, and w is the width, s is the center to center spacing, and $t_{c}$ is the concrete tube thickness, taken as, AofPf, $A_{of}$ is the concrete area enclosed inside the centerline of the FRP jacket, and $P_{f}$ is the perimeter of the area enclosed inside the centerline of the FRP jacket; (5) strengthening scheme; (6) concrete compressive strength; (7) fiber orientation.

A careful examination of the profile of the experimental database presented in Table 2 showed the following remarks: (1) the total number of beams strengthened using EB-FRP tested under significant torsion was 157 beam; (2) although beams are usually connected to a flange (i.e., a floor slab or inverted flanged beam) and the cross section shape have a significant effect on the behavior and design [17,22,35], only 22% of the tested beams had a flanged cross section while 77% investigated rectangular beams; (3) although the full jacket technique is not practical and the U-jacket performs poorly while the usage of anchorage systems for beams with EB-FRP under shear and torsion is gaining a lot of attention [36], less than 20% of the conducted studies investigated using an anchorage; (4) Although FRP continuous jackets can be uneconomic, in most cases, compared to FRP strip jacket, 61% and 37% investigated continuous and strip jackets, respectively; (5) although 45° inclined FRP jackets were found to be the most effective in torsion, only 10% of the experiments examined 45° inclined FRP jackets. In total, the number of tested beams strengthened using fully wrapped strips, continuous fully wrapped, U-jacket, extended U-jacket, and anchored U-jacket were 65, 36, 38, 11, and 13, respectively. Clearly, more work is needed to utilize anchorage devices and extended schemes.

Table 3 shows the parameters investigated by the previous experimental studies. For the case of concrete beams strengthened using EB-FRP, the following parameters were investigated: (1) strengthening scheme that varied depending on the practical application (i.e., access to 3 or 4 faces of the beam); (2) the type of fiber used (CFRP; GFRP, …etc.); (3) fiber orientation (β); (4) original loading and condition before strengthening; (5) number of plies; (6) center to center spacing between strips (s); (7) influence of anchor in U-wrapped test beams; (8) continuous wrap or strips.

## 3. Brief Summaries of Previous Models

### 3.1. FIB

The model developed by FIB [8] adapted the principle of superposition; the total torsion strength is the additive of both the steel and the FRP contribution to the torsion strength of the beam. In other words, the FRP and steel contributions are independent; thus, the FRP torsion contribution ($T_{f}$) was as follows: Where E is Young’s Modulus of the FRP, t is the thickness of the FRP, b and h are the width and depth of the concrete section, respectively, w is the width of the FRP strip, s is the center-to-center spacing between strips, θ is the angle of inclination of the diagonal cracks to the longitudinal axis [7] of the beam. The effective FRP strain ε is being calculated using the following formulas:

(1) ε=0.17(fcm23Eρ)0.3εfu  for CFRP

(2) ε=0.048(fcm23Eρ)0.3εfu for GFRP

where $\varepsilon_{fu}$ is the ultimate strain in the FRP and ρ is FRP reinforcement ratio with respect to concrete calculated as follows:

(3) $$\rho =\frac{2tw}{bs}$$

### 3.2. Deifalla

Deifalla and co-workers [27,28] developed a simple model where the torsion contribution of the FRP contribution ($T_{f}$) can be calculated by the following:

(4) $$T_{f}=\frac{2A_{of}f_{f}A_{f}\left[\cot\beta +\cot\theta \right]\sin\beta }{s},\;\mathrm{for}\;\mathrm{full}\;\mathrm{wrapping}$$

where $A_{of}$ is the area enclosed inside the critical shear flow path due to the strengthening, f is the stress in the FRP sheets at failure, β is the angle of orientation of the fiber direction to the longitudinal axis of the beam, s is the spacing between the centerline of the FRP strips, and $A_{f}$ is the effective area of the FRP-resisting torsion calculated using the follows:

(5) $$A_{f}=ntw\;$$

where n is the number of FRP layers and the FRP effective strain is taken as follows:

(6) $$\varepsilon =\mathrm{minmum}\;\mathrm{of}\;\{\begin{array}{c} \frac{0.33w}{L_{e}s}\\ \frac{0.2\alpha_{f}}{L_{e}}\\ \frac{0.1\varepsilon_{fu}}{{\left(E_{fu}\rho \right)}^{0.86}} \end{array}$$

where the development length ($L_{e}$) is calculated using the following:

(7) $$L_{e}=\sqrt[{2}]{\frac{Et}{\sqrt[{2}]{f_{c}^{\prime }}}}$$

Moreover, $\alpha_{f}$ is a constant to consider the difference in the stress distribution between the continuous FRP sheets and the strips, which is calculated as follows:

(8) $$\alpha_{f}=\sqrt[{2}]{\frac{\left(2-\frac{w}{s\sin\beta }\right)}{\left(1+\frac{w}{s\sin\beta }\right)}}$$

where ρ is FRP reinforcement ratio with respect to concrete calculated as follows:

(9) $$\rho =\frac{A_{f}}{t_{c}s}\;$$

where $t_{c}$ is the thickness of the equivalent hollow tube section taken as AofPf, $A_{of}$ is the concrete area enclosed inside the centerline of the FRP jacket and $P_{f}$ is the perimeter of the area enclosed inside the centerline of the FRP jacket.

### 3.3. ACI

The ACI [10,47] proposed a new design model, where the effective strain for FRP strips in shear is being adapted for this study as follows:

(10) $$\varepsilon_{fmax}=\mathrm{minimum}\;\mathrm{of}\;\{\begin{array}{c} 0.004\;\leq 0.75\varepsilon_{fult}\;\mathrm{for full wrapping}\\ \frac{K_{1}K_{2}L_{e}}{11,900\varepsilon }0.004\;\leq 0.75\varepsilon_{fult}\;\mathrm{for U or side jack} \end{array}$$

(11) K1=(fc 27)23

(12) $$K_{2}=\{\begin{array}{c} \frac{d_{fv}-L_{e}}{d_{fv}}\;\;\;\;\mathrm{for U-jacket}\\ \frac{d_{fv}-2L_{e}}{d_{fv}}\;\;\;\;\mathrm{for side jacket} \end{array}$$

(13) $$L_{e}=\frac{23,300}{{\left(ntE\right)}^{0.58}}\;$$

The selected models are quite similar in the approach used to calculate the torsion capacity, which is based on the hollow tube analogy. However, models vary significantly in the approach used to calculate the design strain and that used to consider different strengthening schemes (i.e., U-jacket, side bond).

## 4. Effects of Significant Parameters on Torsion Gain

**Figure 2:**
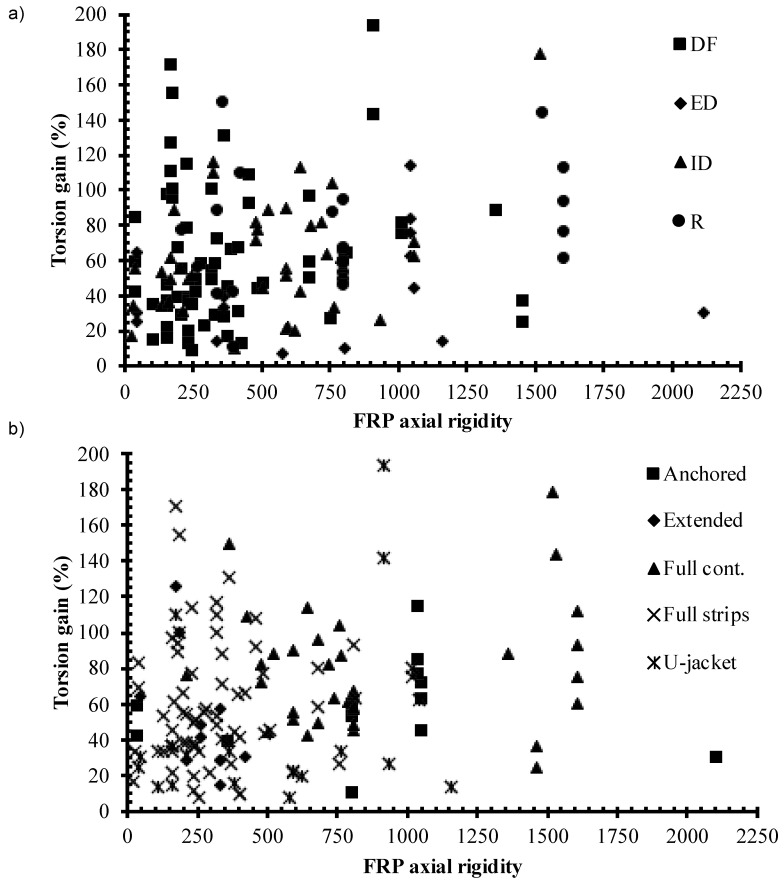
Torsion gain versus FRP axial rigidity (**a**) failure modes and (**b**) strengthening schemes.

In this section, the experimental results from the collected database were implemented to investigate the effects of FRP axial rigidity, strengthening scheme, and failure mode. The axial rigidity of the FRP was calculated as ntE(PcAc), where n is the number of sheet layers, t is the thickness of the FRP sheet, E is the young’s modulus of FRP, $A_{c}$ is the concrete beam cross-section area, and $P_{c}$ is the concrete beam cross-section perimeter. The torsion gain was calculated as the ratio between the torsion contribution of the FRP and the torsion strength of the control beam without FRP. The influence of the FRP axial rigidity on the torsion strength gain is discussed with respect to strengthening schemes used and the observed failure mode. The torsion strength gain is plotted versus the FRP axial rigidity with respect to the failure mode and the strengthening scheme in Figure 2a,b, respectively. In general, the torsion gain increases with the increase in the FRP axial rigidity. The rate of increase is larger for FRP axial rigidity below 1000 compared to that for FRP axial rigidity above 1000. From Figure 2a, at the same FRP axial rigidity and using the same scheme, the percentage of the torsion gain varied significantly, indicating that maybe other parameters might have influenced it. From Figure 2b, it can be seen that data is scattered, which indicates that other parameters have a significant influence. Parameters include but are not limited to the beam dimensions and cross-section shape, and the concrete’s compressive strength and mechanical properties [17,23,29]. Thus, for the machine learning development, the following parameters were selected as follows: (1) X1, is a discrete variable, which represents full wrapping or U wrapping; (2) X2, which represents continuous or strip strengthening; (3) X3 is a discrete variable, which β with 90-, 45-, and 0-degree values; X4 is a discrete variable, which n with a value of 1, 2, and 3; X5 is a continuous variable, which represents the ρ; X6 is a continuous variable, which represents d.

## 5. Machine Learning Models

In recent years, artificial intelligence (AI) has been showing superior results in many applications, such as [59,60,61,62]. It has also shown to have accurate and promising results in structural engineering [63]. From these applications, machine learning (ML) has received exceptional attention from researchers [64,65]. In this paper, 11 machine learning models were implemented. Their performance is evaluated by comparing the accuracy and efficiency of their predictions to the experimentally measured strength. These comparisons are essential for the assessment of such models. All models have been trained using the experimental database, as shown in Section 2. Using 80% of the dataset for training with holdout validation of 15% and testing with the rest of 20%, as shown in Table 4. All models were trained using the same six inputs, and the output was the predicted value of torsion gain. To train an ML model, there are the following four main stages:

- Dividing the database into training and test sets.
- Applying the training methodology for the training set.
- Checking the accuracy requirements.
- Output the predicted values.

A total of 11 machine learning models were developed and used.

### 5.1. Ensembled Trees

An ensemble tree methodology is a weighted combination of multiple regression trees that can provide a strong and accurate prediction. This is because the combining of multiple trees improves the prediction efficiency. Two types of ensemble trees are used, boosted, and bagged, respectively. The boosted tree works as a two-step approach. In the first step, the training dataset is divided into subsets in order to obtain several average-preforming models. Then, in the second step, the maximum performance is obtained by joining all of the models in Step 1 using a defined cost function [66]. The bagged trees create many distinct models by forming bootstraps in a single tree and then integrating them all into one tree. The final decision is obtained as the average of the final trees [67].

### 5.2. Gaussian Process Regression

The Gaussian process regression (GPR) is a Bayesian nonparametric methodology used in solving complex problems. This approach has the ability to provide uncertainty measurements on the predictions. The main advantage of GPR is that the probability distribution is computed over all admissible functions that fit the data in the training set. It also defines a process that which the random variables are tolerated using a Gaussian distribution. Examining squared exponential, Marten 5\2, Exponential and Rational GPR.

### 5.3. Neural Networks

The Artificial Neural Network (ANN) is a supervised learning technique; it was initially inspired by the human biological nervous system. It is a computational black box composed of neurons [68]. ANN is a widely used approach. These methods typically have good predictive accuracy. However, they are not easy to interpret. Model flexibility increases with the size and number of fully connected layers in the neural network. We examined several types of NN model flexibility, narrow, medium, wide, bi-layered, and tri-layered NN, respectively. Each model is a feed-forward, fully connected neural network for classification. The first fully connected layer of the neural network has a connection between the network input, and each subsequent layer has a connection with the previous layer. Each fully connected layer multiplies the input by a weight matrix and then adds a bias vector. An activation function follows each fully connected layer. The final fully connected layer and the subsequent Soft Max activation function produce the network’s output, namely, classification scores and predicted labels.

### 5.4. Results and Discussions

All proposed models were tested using a testing set composed of 20% of the experimental dataset. To evaluate the effectiveness of the proposed models that were reported by comparing the coefficient of determination (*R*^2^), the root mean square error (RMSE), and the mean square error (MAE) for the randomly assigned test set. The three used statistical measures are computed as follows:

(14) $$R^{2}=1-\frac{\sum_{i=1}^{m}{\left(Y_{p}-Y_{o}\right)}^{2}}{\sum_{i=1}^{m}Y_{o}-\frac{1}{m}\sum_{i=1}^{m}Y_{o}}$$

(15) $$\mathrm{RMSE}=\sqrt{\frac{1}{m}\overset{m}{\underset{i=1}{\sum }}{\left(Y_{p}-Y_{o}\right)}^{2}}$$

(16) $$\mathrm{MAE}=\frac{1}{m}\overset{m}{\underset{i=1}{\sum }}\left|Y_{p}-Y_{o}\right|$$

where, $Y_{p}$ is the predicted output and $Y_{o}$ is the real output. While the overfitting potential of the selected models is handled in the following two ways: (1) the dataset random splitting into a training set and a test set; (2) the performance evaluation of the model on the test data, as the model has not been trained on the test data before; therefore, the accuracy of the machine learning models in the training set is an indication of the actual performance of the model on the unseen data. All trained models were trained using a 15-fold cross-validation on the training set using the cross-validated parameters as the hyperparameters of the machine learning model. The optimal parameter for each model is obtained using a 15-fold cross-validation. The results of the proposed models are provided in Table 5. From Table 5, the model that produced the most accurate predictions was the wide neural network model, which reported the highest $R^{2}$, lowest RMSE, and MAE of values 0.93, 1.6634, and 0.98591, respectively. However, it had the longest training time. All proposed models were trained on Intel(R) Core (TM) i5-7200U CPU @ 2.50 GHz, 2.71 GHz, and 16 GB RAM using MATLAB 2021a Statistical and Machine learning toolbox.

**Figure 3:**
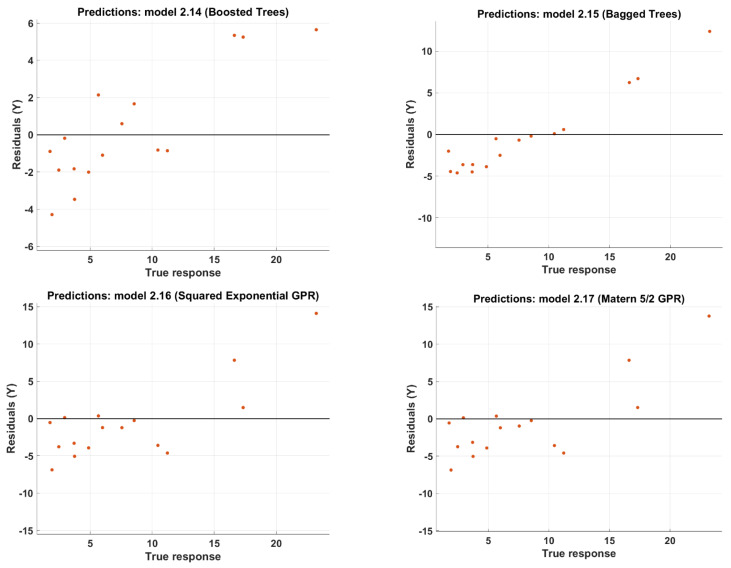

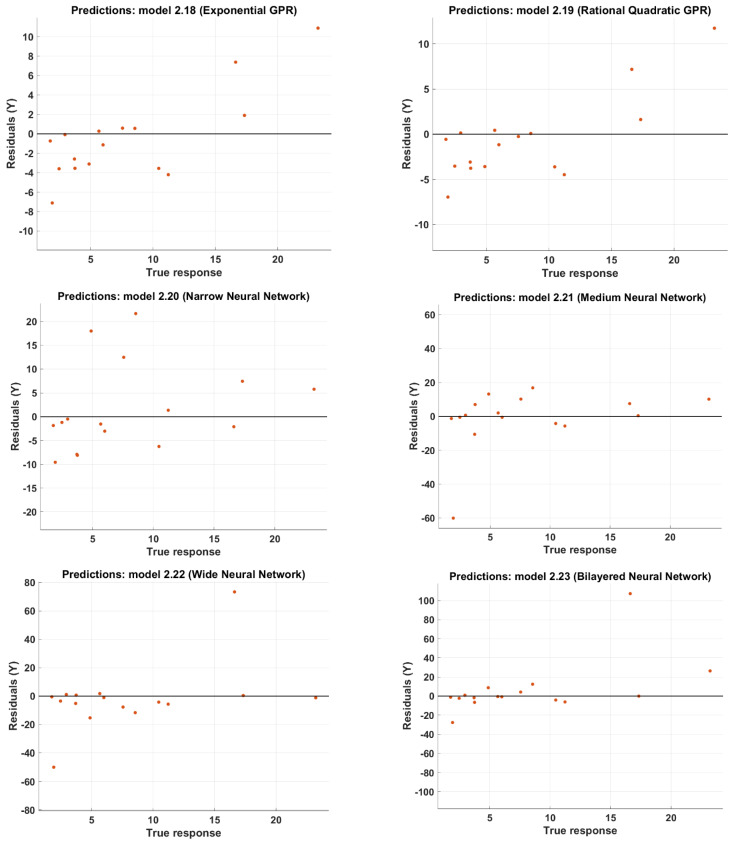

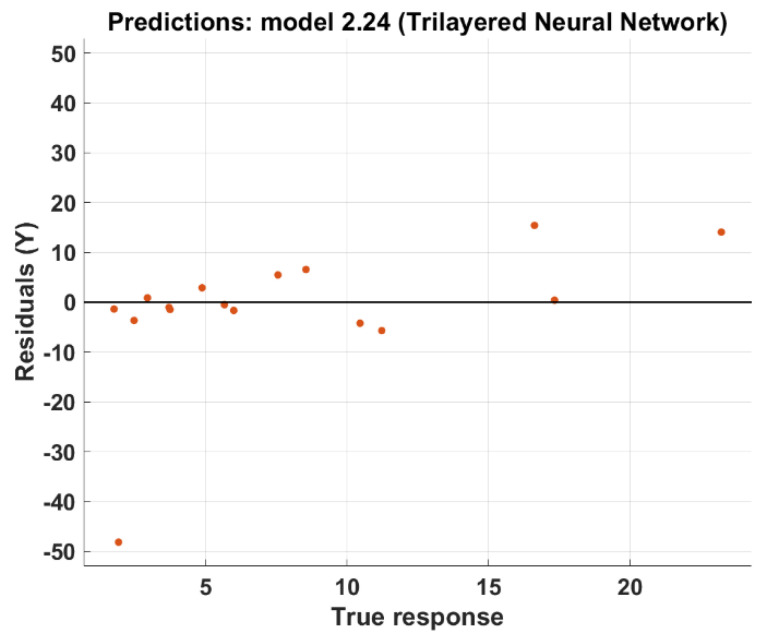
The model predictions for the test set.

Moreover, GPR methods reported the same highest value of $R^{2}$. However, it was not optimal in RMSE and MAE, but generally, it produces acceptable predictions. Figure 3 depicts the scatter distribution of the predictions of the 11 developed ML models. Each model represents the residual plot, where it maps the difference between the observed predicted output and the real output value. The ideal residual plot is also called the null residual plot, where all the data points form an approximately constant width band around the identity horizontal line. The ideal case is to have all points in the linear line, i.e., to have zero error between the predicted and the real output values. The distance between data points and the horizontal line is the error in predictions. In Figure 3, model 1.22 (Wide NN) provides the most accurate predictions with respect to the other ML models developed. Model 1.22 also has the maximum $R^{2}$ and the least RMSE and MAE among the other developed ML models, as depicted in Table 5.

**Figure 4:**
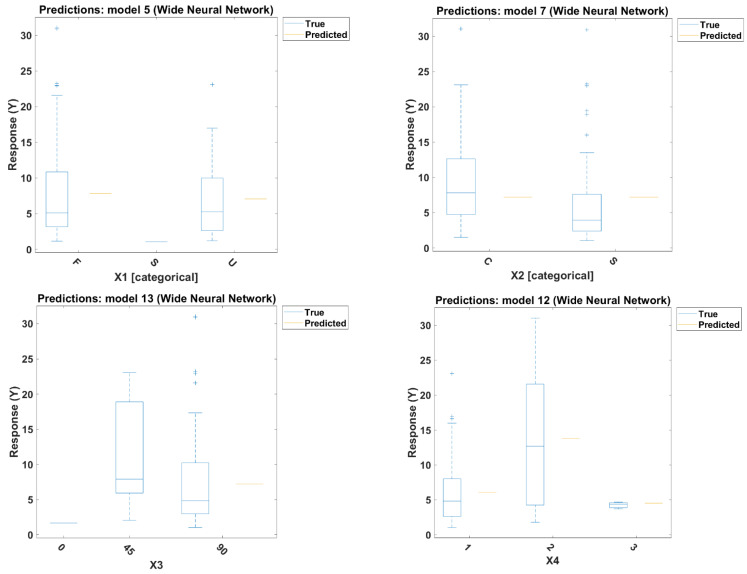

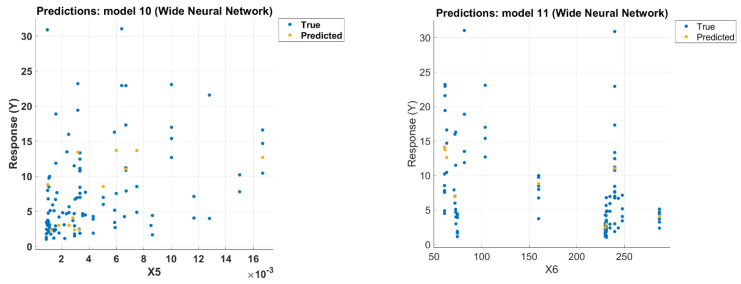
Effect of input parameter on the output prediction.

Moreover, a study of the influence of each of the input parameters on the prediction accuracy is shown in Figure 4. It can be concluded that the most dominant input parameter is X6, followed by X5, then X2, while X3 has proven to have the least effect on the prediction output accuracy. X6 also has the highest factor value of *R*^2^, and the lowest RMSE and MAE in training and testing, respectively.

**Figure 5:**
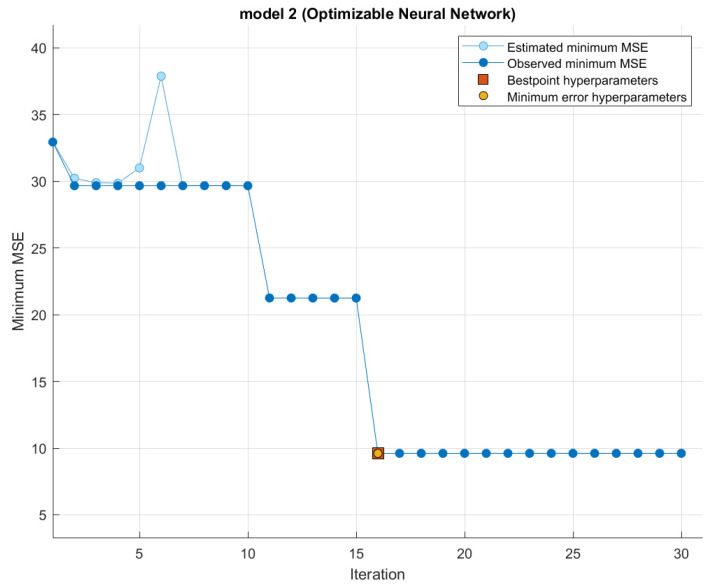
Optimized Wide Neural Network.

Another good visualization for the optimized model is Wide NN; Figure 5 shows how the error decreases as different combinations of hyperparameters are evaluated. The proposed model convergence in the 30th training iteration with the best performance reached in the 16th training iteration and the minimum error hyperparameters. The proposed model is composed of three fully connected layers with sizes equal to 107, 96, and 256 and activation functions ReLU, Tanh, and Sigmoid, respectively, with a regularization strength (Lambda) of value $9.009\times 10^{-8}$ and 900.9009.

## 6. Comparison between Proposed Model and Existing Design Models

**Figure 6:**
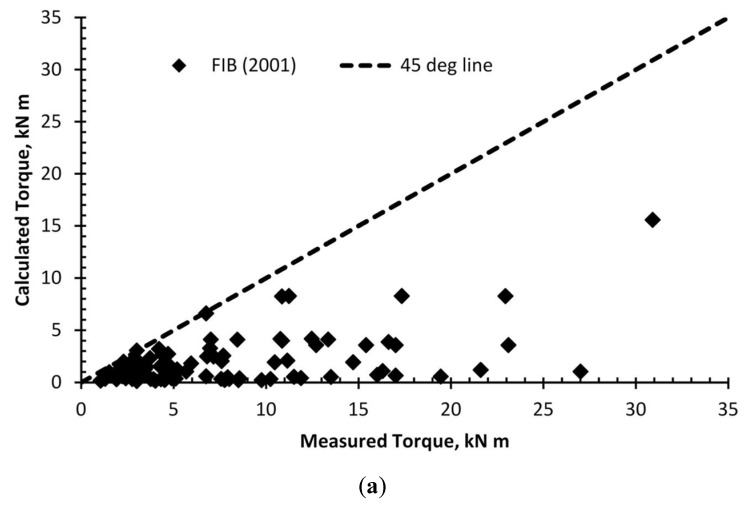

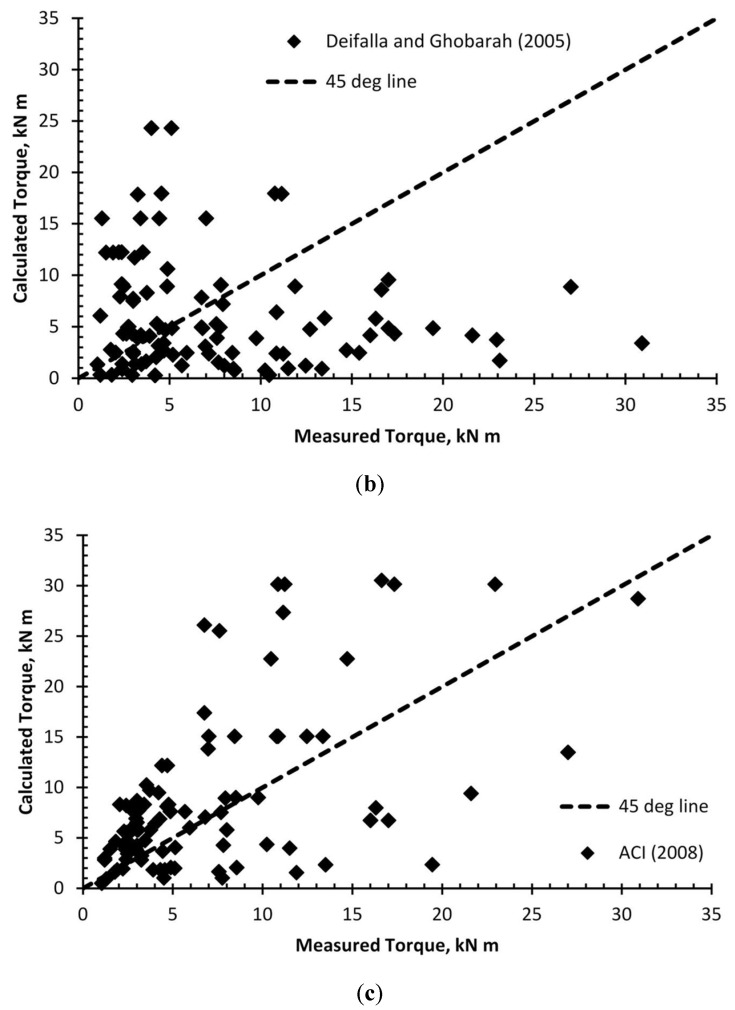
Calculated ultimate strength versus that measured using (**a**) Fib; (**b**) Deifalla and Ghobarah; (**c**) ACI.

The strength was calculated using the available models and a wide neural network model. The angle (ɵ) was taken at 45 degrees to simplify the analysis for the purpose of this study. The ratios between the calculated strength, using the four different models, and the measured strength were graphed as shown in Figure 6. In addition, Table 6 shows the overall average, standard deviation, maximum, minimum, and 95% confidence interval. The proposed model showed a better agreement with the experimental results compared to the other models. Although the ACI (2008) had a comparable average with the wide neural network model, the proposed model predictions were remarkably consistent, having a significantly lower standard deviation compared to the other models. This is due to the ability of the wide neural network to model the true behavior in an accurate and reliable manner.

## 7. Summary and Conclusions

It is clear that the available design models are over-conservative, which is due to the brittle nature of the torsion and FRP. However, the refinement of such models or the development of more accurate and consistent models is the mandate of the research community in order to achieve an economical and safe design.

A total of 11 ML models were developed and tested for the prediction of the torsion gain, including ensembled trees and Gaussian process regression. The selected ML techniques have been widely used in previous studies and are known to effectively analyze different types of datasets. Performance measures were used to evaluate the accuracy of the selected models using *R*^2^, RMSE, and MAE and model training time. The ensembled trees, boosted and bagged, had the worst model performance, with an *R*^2^ of 0.71 and 0.47, respectively.

The most accurate model was the wide neural network model for predicting the torsion strength of concrete beams strengthened using EB-FRP. The models reported the best performance using *R*^2^, RMSE, and MAE with values of 0.93, 16,634 KN, and 0.98 KN, respectively; however, it had the longest training time.

The model was based on an extensive experimental database in order to capture the variation in the following parameters: (1) the strengthening technique; (2) the number and thickness of FRP layers; (3) the spacing between FRP strips; (4) the cross-section dimensions; (5) the FRP type and mechanical properties. The model was verified using an extensive experimental database from various sources and compared with the models available in the literature. In addition, the effect of each parameter on the strength was identified and discussed. Thus, the following conclusions were reached:

- The proposed model based on wide neural networks provides good accuracy and reliable representation of the behavior.
- The most dominant input parameter is effective depth, followed by an FRP reinforcement ratio and then strengthening scheme, while fiber orientation has proven to have the least effect on the prediction output accuracy.
- This study could help further design code development.

## Figures and Tables

**Table 1:** Statistical analysis of experimental database.

	b	d	E	$\rho_{f}$	$f_{c}^{\prime }$	*β*	Torsion Gain
	(mm)	(mm)	(MPa)	(%)	(MPa)	(Degrees)	(kN m)
Overall							
Min.	150	90	61	0.09	25	0	1.04
Max.	400	500	287	1.67	80.56	90	31.05
Avg.	288	155	178	0.42	37.48	85	8
SD	74.6	48.6	80	0.39	15.70	15	6
C.O.V.	26%	31%	45%	92%	42%	18%	85%

**Table 2:** Profile of previous experimental results.

Training or Testing	Study	No. of Beams	Shape				$f_{c}^{\prime }$ < 40	$f_{c}^{\prime }$ ≥ 40	h ≤ 400 mm	GFRP	CFRP	Full	U-Jacket	Side	Top or Bottom	Strips	Continuous	*β*		
			Rectangle	T-Shaped	L-Shaped	Hollow												0	45	90
Data set used for training	[37]	9	9	-	-	-	9	-	9		9	7	-	2	1	7		3	-	7
	[38]	4	4	-	-	-	4	-	4	4		4	-	-	-	-	4	-	-	4
	[39]	8	8	-	-	-	8	-	8	2	6	8	-	1	-	5	2	-	-	6
	[40]	7	7	-	-	-	7	-	7	7	-	4	3	-	-	4	3	5	-	2
	[41]	7	7	-	-	-	7	-	7	-	7	5	2	-	-	5	2	4	-	5
	[42]	1	1	-	-	-	-	-	1	-	1	1	-	-	-	-	1	-	-	
	[43]	4	-	-	4	-	4	-	4	-	-	-	4	-	-	-	4	2	1	3
	[44]	6	6	-	-	-	6	-	6	3	3	2	-	4	-	6		2		5
	[45]	6	6	-	-	-	6	-	6		6	6	-	-	-	3	3	-	-	6
	[46]	2	2	-	-	-		2	2		2	2	-	-	-	2	-	-	-	2
	[23,27,28,29]	4	-	4	-	-	4	-	4	-	-	2	2	-	-	-	4	-	4	-
	[46]	4	1	-	-	3	4	-	4	-	4	4		-	-	4	-	-	-	-
	[47]	10	10	-	-		10	-	10	5	5	6	4	-	-	4	6	-	-	10
	[48]	16	10	3	3		16	-	16	14	2	8	8	-	-	14	2	1	2	14
	[49]	8	6	2	-	-	8	-	8	-	8	6	2	-	-	3	5	-	-	8
	[50]	10	10	-	-	-		10	10	-	10	8	2	-	-	2	8	-	-	10
	[51]	2	2	-	-	-	2		2	2	-	-	2	-	-	2		-	-	2
	[52]	7	7	-	-	-	-	7	7	-	7	6	1	-	-	2	5	-	-	7
	[53]	8	8	-	-	-	8	-	8	-	8	6	2	-	-	3	5	-	-	8
	[54]	4	-	4	-	-	3	-	3		4	-	4	-	-	4	-	-	1	3
Dataset for testing	[55]	8	8		-	-	8	-	8	8		8	-	-	-	6	2	-	2	6
	[22]	8	2	2	4		8	-	8	-	8	3	5	-	-	8	-	-	3	5
	[56]	7	-	-	7		7	-	7	-	7	2	5	-	-	7	-	-	-	7
	[57]	1	1	-	-	-	1	-	1	1	-	1	-	-	-	1	-	-	-	1
	[58]	6	6	-	-	-	6	-	6	6	-	6	-	-	-	4	2	1	2	3
	Number of tested beams	157	121	15	18	3	136	19	156	52	97	105	46	7	1	96	58	18	17	124
	Percentage (%)		77	10	11	2	87	12	99	33	67	67	29	4	1	61	37	11	11	79

**Table 3:** Parameters investigated by various researchers.

Study	Anchors	Spacing	Preloading	Plies	Size Effect
[37]					
[38]					
[39]					
[40]					
[41]					
[42]					
[43]					
[44]					
[45]					
[46]					
[23,27,29]					
[46]					
[47]					
[48]					
[49]					
[50]					
[51]					
[52]					
[53]					
[54]					
[55]					
[22]					
[56]					
[57]					
[58]					
Number of studies	7	9	4	12	2
Percentage (%)	28%	36%	16%	48%	8%

**Table 4:** Statistical analysis of training and validating set.

	b	d	E	$\rho_{f}$	$f_{c}^{\prime }$	*β*	Torsion Gain
	mm	mm	MPa	(%)	MPa	Degrees	kN m
Training set							
Min.	150	90	61	0.1	45	25	1.04
Max.	400	500	287	1.7	90	80.56	30.9
Avg.	297	160	182	0.5	87	40	8
SD	74	53	80	0.4	11	17	6
C.O.V.	25%	33%	44%	91%	13%	42%	76%
Validation set							
Min.	150	90	61.58	0.09	0	25	1.67
Max.	400	150	232	0.86	90	32	31.05
Avg.	258	139	165	0.29	79	28	8
SD	69	17	80	0.24	24	3	8
C.O.V.	27%	12%	49%	82%	30%	11%	111%

**Table 5:** Results comparison between ML methods.

Models	R-Squared	RMSE	MAE	Training Time (s)
Ensemble Trees				
Boosted	0.71	3.4274	2.1631	2.7569
Bagged	0.47	4.6322	3.3205	3.1841
Gaussian Process Regression				
Squared Exponential	0.93	1.6854	1.1702	5.2342
Marten 5/2	0.93	1.6778	2.8149	1.175
Exponential	0.93	1.7447	1.1914	1.9939
Rational Quadratic	0.93	1.6863	1.1523	4.8316
Neural Network				
Narrow	0.56	1.6854	2.8724	3.1006
Medium	0.92	1.6778	1.1643	1.175
Wide	0.93	1.6634	0.98591	7.7472
Bi-layered	0.71	3.4655	2.2849	2.7611
Tri-layered	0.92	1.8611	3.4636	3.7419

**Table 6:** Comparing ratio of measured and predicted torsion strength from various models.

Models	FIB (2001)	Deifalla and Ghobarah (2005)	ACI (2008)	Wide Neural NetWork Model
Average	8.50	2.31	1.22	1.11
Standard Deviation	9.48	3.58	1.53	0.40
Maximum	42.29	22.62	8.25	2.32
Minimum	0.89	0.21	0.07	0.23
Lower 95%	6.47	1.55	0.89	1.02
Upper 95%	10.52	3.08	1.54	1.20

## Data Availability

All developed data are included in the paper.
